# Supplementary material for: Genetic comparisons of interleukin-17 reveal a framework for complex signaling evolution
Source: bioRxiv. 2026 Apr 14:2026.04.13.718218. Preprint. [Version 1] doi: 10.64898/2026.04.13.718218 (PMC13105070; doi:10.64898/2026.04.13.718218)
Supplement: Supplement 3 [file media-3.zip › Supplemental files for paper/ERC/IL17xIL17_comparisons/IL17C_IL17RC.pdf]

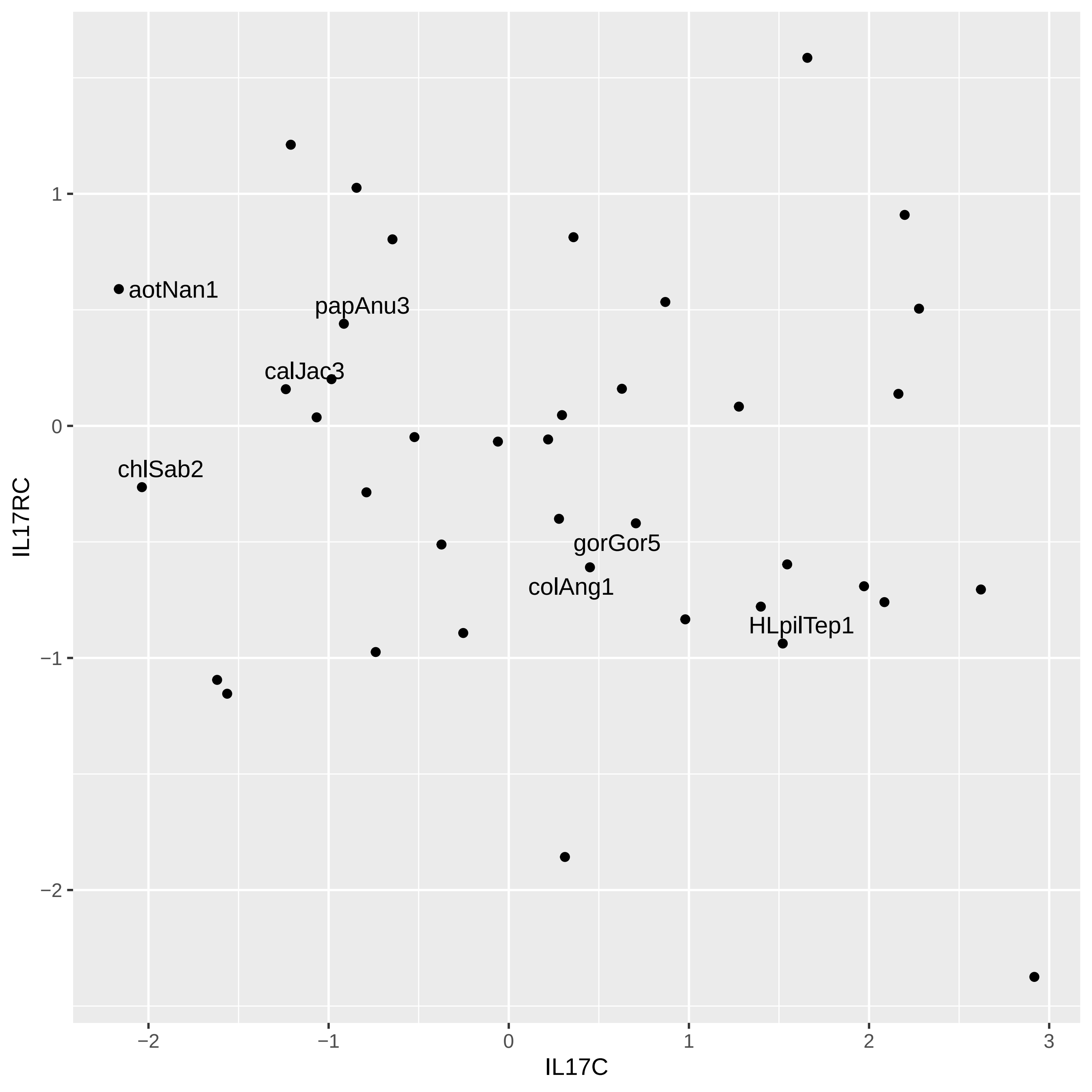

aotNan1
papAnu3
calJac3
chlSab2
gorGor5
colAng1
HLpilTep1
1
0
−1
−2
−2
−1
0
1
2
3
IL17RC
IL17C
